# Supplementary material for: The Roles of CCCH Zinc-Finger Proteins in Plant Abiotic Stress Tolerance
Source: Int J Mol Sci. 2021 Aug 3;22(15):8327. doi: 10.3390/ijms22158327 (PMC8347928; doi:10.3390/ijms22158327)
Supplement: Supplementary file 1 [file ijms-22-08327-s001.zip › ijms-1285258-supplementary.pdf]

Supplementary Table S1. Functions of CCCH proteins in abiotic stress responses and mode of actions

Gene	Abiotic stresses and mode of actions							CCCH motif type	References
	Salt	Drought	Cold	Flooding	Oxidative	Transcriptional DNA binding	Post-transcriptional RNA binding		
OsC3H33	+							C-X_8-C-X_5-C-X_3-H	[1]
OsC3H37	+							C-X_8-C-X_5-C-X_3-H	[1]
OSC3H47	+	+					+	C-X_7-C-X_5-C-X_3-H	[2]
OSC3H50	+							C-X_8-C-X_5-C-X_3-H	[1]
AtZFP1	+					+		C-X_8-C-X_5-C-X_3-H	[3]
GhZFP1	+	+						C-X_8-C-X_5-C-X_3-H and C-X_5-C-X_4-CX_3-H	[4]
AtSZF1	+					+		C-X_7-C-X_5-C-X_3-H	[5]
AtSZF2	+					+		C-X_7-C-X_5-C-X_3-H	[5]
AtOZF1					+	+		C-X_{12}-C-X_{10}-C-X_3-H, C-X_7-C-X_5-C-X_3-H and C-X_5-C-X_4-C-X_3-H	[6]
AtOZF2	+							C-X_{12}-C-X_{10}-C-X_3-H, C-X_7-C-X_5-C-X_3-H and C-X_5-C-X_4-C-X_3-H	[7]
OsTZF1	+						+	C-X_8-C-X_5-C-X_3-H	[8]
OsC3H10		+				+		C-X_{10}-C-X_5-C-X_3-H and C-X_5-C-X_4-C-X_3-H	[9]

OsTZF5	+					+	C-X$_8$-C-X$_5$-C-X$_3$-H and C-X$_5$-C-X$_4$-C-X$_3$-H	[10]
BoC3H	+				+		C-X$_7$-C-X$_5$-C-X$_3$-H and C-X$_5$-C-X$_4$-C-X$_3$-H	[11]
AtC3H17	+						C-X$_7$-C-X$_5$-C-X$_3$-H	[12]
AtTZF1		+	+				C-X$_8$-C-X$_5$-C-X$_3$-H	[13]
AtTZF2		+					C-X$_8$-C-X$_5$-C-X$_3$-H	[14]
AtTZF3		+					C-X$_8$-C-X$_5$-C-X$_3$-H	[14]
GhTZF1		+			+		C-X$_7$-C-X$_5$-CX$_3$-H and C-X$_5$-C-X$_4$-CX$_3$-H	[15]
AetTZF1		+			+		C-X$_7$-CX$_5$-C-X$_3$-H and C-X$_5$-C-X$_4$-C-X$_3$-H	[16].
OsCCCH-Zn-1				+			C-X$_8$-C-X$_5$-C-X$_3$-H	[17]
OsCCCH-Zn-2				+			C-X$_8$-C-X$_5$-C-X$_3$-H	[17]
OsCCCH-Zn-3				+			C-X$_8$-C-X$_5$-C-X$_3$-H	[17]
PvC3H72			+		+		C-X$_8$-C-X$_5$-C-X$_3$-H	[18]
IbC3H18	+	+			+		C-X$_7$-C-X$_5$-C-X$_3$-H	[19]
PeC3H74	+						C-X$_8$-C-X$_5$-C-X$_3$-H and C-X$_7$-C-X$_5$-C-X$_3$-H	[20]
DgC3H1			+		+		C-X$_{5-14}$-C-X$_{4-5}$-C-X$_3$-H	[21]

References

1. Jamil, M.; Iqbal, W.; Bangash, A.; Rehman, S. U.; Imran, Q. M.; Rha, E. S., Constitutive expression of OSC3H33, OSC3H50 and OSC3H37 genes in rice under salt stress. *Pak. J. Bot* 2010, 42, (6), 4003-4009.
2. Wang, W.; Liu, B.; Xu, M.; Jamil, M.; Wang, G., ABA-induced CCCH tandem zinc finger protein OsC3H47 decreases ABA sensitivity and promotes drought tolerance in Oryza sativa. *Biochemical and Biophysical Research Communications* 2015, 464, (1), 33-37.
3. Han, G.; Wang, M.; Yuan, F.; Sui, N.; Song, J.; Wang, B., The CCCH zinc finger protein gene AtZFP1 improves salt resistance in Arabidopsis thaliana. *Plant molecular biology* 2014, 86, (3), 237-253.
4. Guo, Y. H.; Yu, Y. P.; Wang, D.; Wu, C. A.; Yang, G. D.; Huang, J. G.; Zheng, C. C., GhZFP1, a novel CCCH-type zinc finger protein from cotton, enhances salt stress tolerance and fungal disease resistance in transgenic tobacco by interacting with GZIRD21A and GZIPR5. *New Phytologist* 2009, 183, (1), 62-75.
5. Sun, J.; Jiang, H.; Xu, Y.; Li, H.; Wu, X.; Xie, Q.; Li, C., The CCCH-type zinc finger proteins AtSZF1 and AtSZF2 regulate salt stress responses in Arabidopsis. *Plant and Cell Physiology* 2007, 48, (8), 1148-1158.
6. Huang, P.; Chung, M.; Ju, H.; Na, H.; Lee, D. J.; Cheong, H.; Kim, C. S., Physiological characterization of the Arabidopsis thaliana Oxidation-related Zinc Finger 1, a plasma membrane protein involved in oxidative stress. *Journal of Plant Research* 2011, 124, (6), 699-705.
7. Huang, P.; Ju, H.; Min, J.; Zhang, X.; Chung, J.; Cheong, H.; Kim, C. S., Molecular and Physiological Characterization of the Arabidopsis thaliana Oxidation-Related Zinc Finger 2, a Plasma Membrane Protein Involved in ABA and Salt Stress Response Through the ABI2-Mediated Signaling Pathway. *Plant and Cell Physiology* 2012, 53, (1), 193-203.
8. Jan, A.; Maruyama, K.; Todaka, D.; Kidokoro, S.; Abo, M.; Yoshimura, E.; Shinozaki, K.; Nakashima, K.; Yamaguchi-Shinozaki, K., OsTZF1, a CCCH-tandem zinc finger protein, confers delayed senescence and stress tolerance in rice by regulating stress-related genes. *Plant Physiology* 2013, 161, (3), 1202-1216.
9. Seong, S. Y.; Shim, J. S.; Bang, S. W.; Kim, J. K., Overexpression of OsC3H10, a CCCH-Zinc Finger, Improves Drought Tolerance in Rice by Regulating Stress-Related Genes. *Plants (Basel, Switzerland)* 2020, 9, (10).
10. Selvaraj, M. G.; Jan, A.; Ishizaki, T.; Valencia, M.; Dedicova, B.; Maruyama, K.; Ogata, T.; Todaka, D.; Yamaguchi-Shinozaki, K.; Nakashima, K.; Ishitani, M., Expression of the CCCH-tandem zinc finger protein gene OsTZF5 under a stress-inducible promoter mitigates the effect of drought stress on rice grain yield under field conditions. *Plant Biotechnol J* 2020, 18, (8), 1711-1721.
11. Jiang, M.; Jiang, J.; Miao, L.; He, C., Over-expression of a C3H-type zinc finger gene contributes to salt stress tolerance in transgenic broccoli plants. *Plant Cell Tissue and Organ Culture* 2017, 130, (2), 239-254.
12. Seok, H.; Nguyen, L. V.; Park, H.; Tarte, V. N.; Ha, J.; Lee, S.; Moon, Y., Arabidopsis non-TZF gene AtC3H17 functions as a positive regulator in salt stress response. *Biochemical and Biophysical Research Communications* 2018, 498, (4), 954-959.
13. Lin, P. C.; Pomeranz, M. C.; Jikumaru, Y.; Kang, S. G.; Hah, C.; Fujioka, S.; Kamiya, Y.; Jang, J. C., The Arabidopsis tandem zinc finger protein AtTZF1 affects ABA-and GA-mediated growth, stress and gene expression responses. *The Plant Journal* 2011, 65, (2), 253-268.

14. Lee, S.-j.; Jung, H. J.; Kang, H.; Kim, S. Y., Arabidopsis Zinc Finger Proteins AtC3H49/AtTZF3 and AtC3H20/AtTZF2 are Involved in ABA and JA Responses. *Plant and Cell Physiology* 2012, 53, (4), 673-686.

15. Zhou, T.; Yang, X.; Wang, L.; Xu, J.; Zhang, X., GhTZF1 regulates drought stress responses and delays leaf senescence by inhibiting reactive oxygen species accumulation in transgenic Arabidopsis. *Plant molecular biology* 2014, 85, (1-2), 163-177.

16. Jiang, A.-L.; Xu, Z.-S.; Zhao, G.-Y.; Cui, X.-Y.; Chen, M.; Li, L.-C.; Ma, Y.-Z., Genome-wide analysis of the C3H zinc finger transcription factor family and drought responses of members in Aegilops tauschii. *Plant molecular biology reporter* 2014, 32, (6), 1241-1256.

17. Pandey, D. M.; Kim, S., Identification and expression analysis of hypoxia stress inducible CCCH-type zinc finger protein genes in rice. *Journal of Plant Biology* 2012, 55, (6), 489-497.

18. Xie, Z.; Lin, W.; Yu, G.; Cheng, Q.; Xu, B.; Huang, B., Improved cold tolerance in switchgrass by a novel CCCH-type zinc finger transcription factor gene, PvC3H72 , associated with ICE1–CBF–COR regulon and ABA-responsive genes. *Biotechnology for Biofuels* 2019, 12, (1), 224.

19. Zhang, H.; Gao, X.; Zhi, Y.; Li, X.; Zhang, Q.; Niu, J.; Wang, J.; Zhai, H.; Zhao, N.; Li, J., A non-tandem CCCH-type zinc-finger protein, IbC3H18, functions as a nuclear transcriptional activator and enhances abiotic stress tolerance in sweet potato. *New Phytologist* 2019, 223, (4), 1918-1936.

20. Chen, F.; Liu, H.-L.; Wang, K.; Gao, Y.-M.; Wu, M.; Xiang, Y., Identification of CCCH Zinc Finger Proteins Family in Moso Bamboo (Phyllostachys edulis), and PeC3H74 Confers Drought Tolerance to Transgenic Plants. *Frontiers in plant science* 2020, 11, 1697.

21. Bai, H.; Lin, P.; Li, X.; Liao, X.; Wan, L.; Yang, X.; Luo, Y.; Zhang, L.; Zhang, F.; Liu, S., DgC3H1, a CCCH zinc finger protein gene, confers cold tolerance in transgenic chrysanthemum. *Scientia Horticulturae* 2021, 281, 109901.
